# The first euthemistid damsel-dragonfly from the Middle Jurassic of China (Odonata, Epiproctophora, Isophlebioptera)

**DOI:** 10.3897/zookeys.261.4371

**Published:** 2013-01-24

**Authors:** Yongjun Li, André Nel, Chungkun Shih, Dong Ren, Hong Pang

**Affiliations:** 1 State Key Laboratory of Biocontrol and Institute of Entomology / Key Laboratory of Biodiversity Dynamics and Conservation of Guangdong Higher Education Institutes Sun Yat-Sen University, Guangzhou, China; 2 CNRS UMR 7205, CP 50, Entomologie, Muséum National d’Histoire Naturelle, 45 rue Buffon, F-75005 Paris, France; 3 College of Life Science, Capital Normal University, 105 Xisanhuanbeilu, Haidian District, Beijing 100048 China

**Keywords:** Euthemistidae, Sphenophlebiidae, gen. et sp. n., Middle Jurassic, China, mixture of characters

## Abstract

*Sinoeuthemis daohugouensis* **gen. et sp. n.** is the first record of the isophlebiopteran family Euthemistidae from Middle Jurassic of northeast China, while previously this family was restricted to the early Late Jurassic Kazakhstan. This new finding allows us to emend the family diagnosis with hindwing characters. This new species shows a mixture of characters alternatively present in different genera of the two families Euthemistidae and Sphenophlebiidae.

## Introduction

The Mesozoic Isophlebioptera Bechly, 1996 is a very large clade subdivided into four subgroups: Euthemistidae, Parazygoptera, Selenothemistidae, and Isophlebiida. The family Euthemistidae Pritykina, 1968 comprises one genus and two species (both based on forewings). Our knowledge on the Euthemistidae remains indigent and its exact position is somewhat uncertain, even if it is probably very inclusive in the Isophlebioptera. Here we describe a new genus and a new species collected from the Jiulongshan Formation, Middle Jurassic of Daohugou, Inner Mongolia, China, where abundant well-preserved fossil insects are found, including 19 reported orders so far, and some of the oberservations are of great importance (e.g. Ren et al. 2009, 2010a, 2010b, 2010c; Gao et al. 2012; Gu et al. 2012; Shi et al. 2011). The new genus is closely related to the genus *Euthemis* Pritykina, 1968 and for the first time we show the hindwing characters of the family Euthemistidae. The new fossil has a mixture of characters alternatively present in different genera of the two families Euthemistidae and Sphenophlebiidae. Therefore, this new finding is of great importance for future clarification of the relationships between the Euthemistidae, Sphenophlebiidae, and other Parazygoptera. Furthermore, the new specimen expands the age and distribution of Euthemistidae from early Late Jurassic Karatau (Kazakhstan) to Middle Jurassic northeast China.

## Material and method

The study is based on one specimen (No. CNU-ODO-NN2012004, positive imprint and negative imprint) housed in the Key Laboratory of Insect Evolution and Environmental Changes, Capital Normal University, Beijing, China. The specimen was examined with a Leica MZ12.5 dissecting microscope and illustrated with the aid of a drawing tube attached to the microscope. Line drawings were made using Adobe Photoshop CS graphic software.

We use the following standard abbreviations: AA, anal vein; AP, anal posterior; Ax0 Ax1 Ax2, primary antenodal cross-veins; CuAa, distal branch of cubitus anterior; CuAb, proximal branch of cubitus anterior; IR1, IR2, intercalary radial veins; MAa, distal branch of median anterior; MAb, posterior branch of median anterior; MP, median posterior; N, nodus; O, oblique vein; Pt, pterostigma; RA, radius anterior; and RP, radius posterior; ScP, subcosta posterior. We follow the taxonomy of Isophlebiida indicated by the phylogenetic system of Bechly (1996), but do not accept all the synapomorphies he proposed (see discussion below).

## Systematic palaeontology

### Order Odonata Fabricius, 1793

Clade Isophlebioptera Bechly, 1996

Superfamily Isophlebioidea Handlirsch, 1906

#### Family Euthemistidae

Pritykina, 1968

http://species-id.net/wiki/Euthemistidae

##### Genus included.

type genus *Euthemis* Pritykina, 1968 and*Sinoeuthemis* gen. n.

##### Emended familial diagnosis.

Several long intercalary veins between IR1 and RP1, and between IR1 and RP2, as well as between RP3/4 and IR2, and between IR2 and RP2 (intercalaries parallel to main longitudinal veins without apparent origin on them, but originating in cross-venation); extremely narrow postdiscoidal area (in forewings and probably also in hindwings); not petiolate; numerous secondary antenodal crossveins between anterior wing margin and ScP distal of Ax2; discoidal cells opened in forewing and closed in hindwing; hindwing subdiscoidal area transverse, posteriorly closed, short and broad, with vein CuAb makes a strong angle with AA; hindwing gaff (basal CuA before its branching) not very long; RP2 aligned with subnodus; crossveins in hindwing postdiscoidal space are not very long and not oblique.

#### Genus Sinoeuthemis gen. n.

urn:lsid:zoobank.org:act:1DE29A69-7318-4D84-B0EE-209593108C8A

http://species-id.net/wiki/Sinoeuthemis

##### Diagnosis.

Wings relatively short and very short CuA with weak posterior branches in both fore and hindwings.

##### Etymology.

Named after *Sinica*, Latin name for China and *Euthemis*, the type genus. Gender feminine.

#### Sinoeuthemis daohugouensis sp. n.

urn:lsid:zoobank.org:act:6783C7B8-8791-48B0-9722-010427CECC6E

http://species-id.net/wiki/Sinoeuthemis_daohugouensis

Figure 1

##### Material.

Holotype specimen No. CNU-ODO-NN2012004.

##### Diagnosis.

As for the genus.

##### Description.

**Figure 1.**
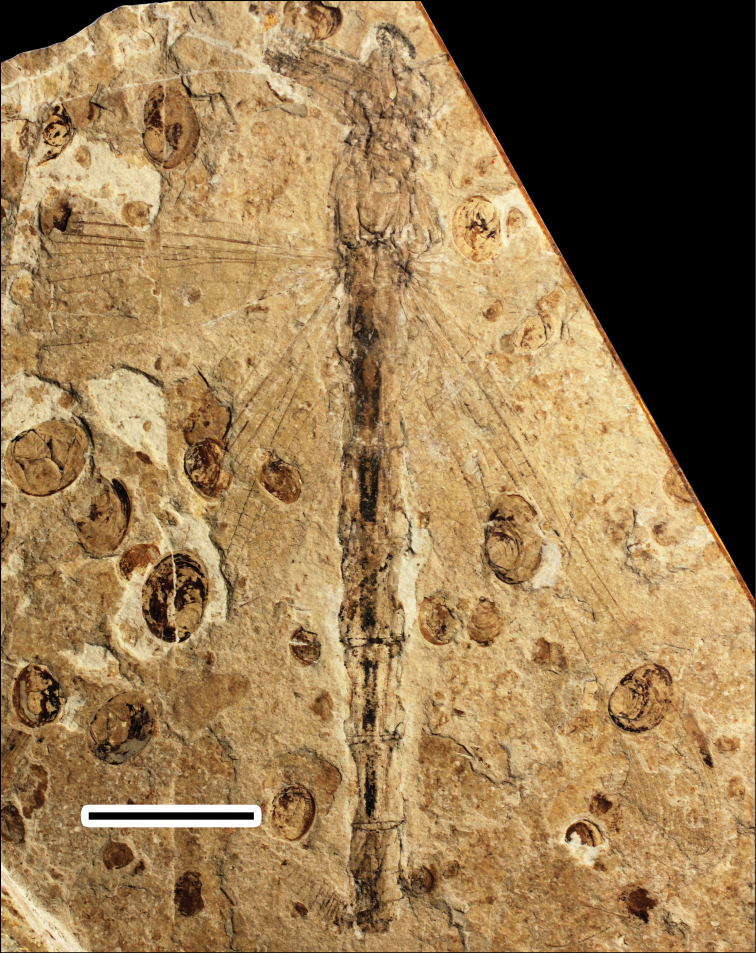
Photo of positive imprint of CNU-ODO-NN2012004. Scale bar represents 10 mm.

**Figure 2.**
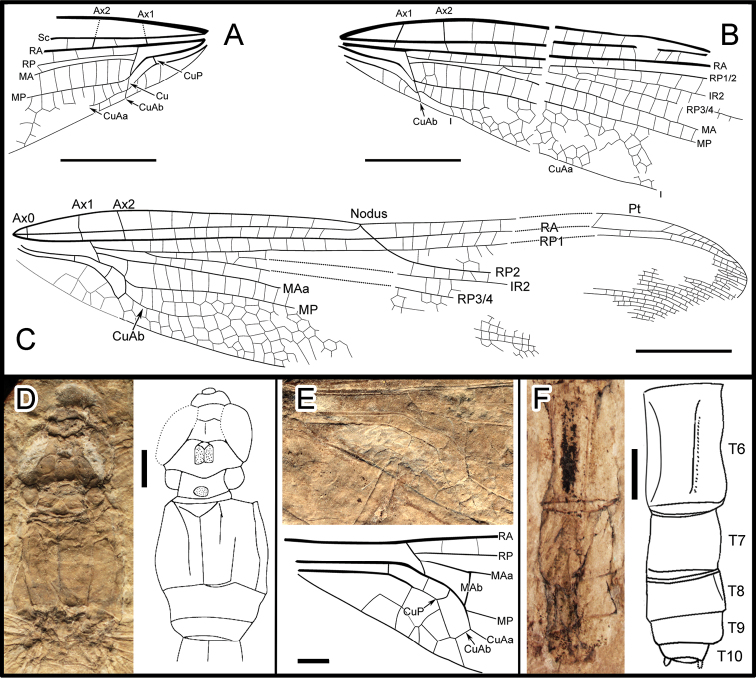
**A, B** Drawing of forewings, from negative imprint **C** Drawing of hindwing, from positive imprint, combined with anal area of the negative imprint (dotted line) **D** Detail photo and drawing of head and thorax, from negative imprint **E** Detail photo and drawing of hindwing anal area, from negative imprint **F** Detail photo and drawing of the lower part of abdomen, from positive imprint. Scale bars represent 5 mm in **A–C** and 1 mm in **D–F**.

A body with a thorax, abdomen, head, two legs and forewings and hindwing articulated. **Body** (Fig. 1; Fig. 2D, F) 53.0 mm long (from head to anal appendages); head 5.1 mm long, 5.2 mm wide, with broad eyes, 1.7 mm long, well separated, 1.0 mm apart in the mid level; thorax about 8 mm long, max width 6.5 mm; abdomen about 3.8 mm wide in the mid part, slightly narrowed at the end; cercus and epiproct very short; there is no secondary genital structure on segment 2 and anal area rounded (female). **Forewing** (based on negitive imprint, two forewing fragments combined; Fig. 2A, B), preserved with basal half, 22.4 mm long; no petiole (AA and AP separate at wing base); one row of cells between posterior wing margin and AA; AA parallel to MP + Cu; median and submedian areas free; a curved strong vein CuP between submedian and subdiscoidal areas, in a distal position just basal of arculus; subdiscoidal space free of cross-veins, transverse; discoidal space basally opened; RP+MA nearly straight, separated at nearly a right angle from RA in arculus; distance between base of RP and point of separation between MAa and MAb 0.4 mm, RP and MA well parallel; MAb 0.9 mm long, well aligned with distal free part of CuA; CuA separates from MP 4.3 mm from wing base and directed towards posterior wing margin for 0.6 mm; distal free part of CuA strong, CuA distally fused with AA; CuA divided into a very short CuAb directed towards posterior wing margin and CuAa basally more or less parallel to posterior wing margin and distally delimitating a short and narrow cubito-anal area, with 1-2 posterior branches and 1-2 rows of cells at its broadest part; apex of CuA slightly distal level of base of RP3/4; area between CuA and MP with one row of cells; distal of apex of CuA, area between MP and posterior wing margin very long and broad; MP nearly straight, certainly reaching posterior wing margin well distal of nodus level; MAa more or less parallel with MP, nearly straight in its preserved part; postdiscoidal area with one row of cells, 1.0 mm wide near discoidal cell and narrowing distally; Ax0 not preserved; Ax1 0.6 mm basal of arculus, disposed obliquely to ScP and R + MA, Ax2 2.4 mm distal of arculus, with inverted obliquity; eight preserved secondary antenodal cross-veins between C and ScP distal of Ax2; 13 visible secondary antenodal cross-veins between ScP and RA distal of Ax2; 15 preserved cross-veins in area between RA and RP between arculus and subnodus; base of RP3/4 5.0 mm distal of arculus, closer to arculus than to nodus; base of IR2 close to that of RP3/4, 3.8 mm distally; no visible antefurcal cross-vein in space between RP and MA basal of midfork (base of RP3/4); nodal structures not preserved; area between MA and RP3/4 narrow basally but distally widening. **Hindwing** (mainly based on right hindwing of positive imprint, combined with anal area of left hindwing on negitive imprint; Fig. 2C, E) hyaline, more complete than forewing, 39.7 mm long, estimate 8 mm wide at the level of nodus; primary antenodal crossveins Ax0, Ax1 and Ax2 are well preserved; Ax1 nearly perpendicular to ScP; Ax2 slightly oblique; distance form base to Ax1 3.6 mm, to arculus 4.2 mm, to nodus 18.7 mm; distance from arculus to the first fork of RP 2.8 mm; Ax1 0.4 mm basal of arculus and Ax2 1.9 mm distal of arculus; nine secondary antenodal cross-veins between C and ScP, but 11 secondary antenodal cross-veins between ScP and RA, distal of Ax2; no petiole; anal area about 6 mm long, 1.7 mm wide, rather rounded elongate in shape, with two rows of irregular cells between AA and AP; no anal angle; no membranule; AA distally strongly bent towards posterior wing margin and nearly parallel with MP + CuA, distally fused with CuAb; median and submedian areas free; curved vein CuP slightly basal of arculus; subdiscoidal area transverse, posteriorly closed, short and broad, with one cross-vein, 1.3 mm long, 0.8 mm wide; discoidal cell basally closed, 1.6 mm long, 0.5 mm wide, free of cross-veins, length of proximal side, 0.8 mm; RP + MA separates at approximately a right angle from RA and strongly curved in arculus; RP separated from MA 0.3 mm distally; just distal of arculus base, MA basally strong and divided into MAa and MAb distally; MAb short, 0.8 mm long, aligned with distal free part of CuA; MP + CuA separated into MP and CuA at distal end of MAb; distal free part of CuA strong, separates from MP 6.1 mm from wing base and extends towards posterior wing margin for 0.7 mm; CuA distally divided into CuAa and CuAb, CuAb short, 0.5 mm long, extending towards basal wing margin and meets main branch of AA; CuAa basally more or less parallel to posterior wing margin with two rows of cells between them; CuAa short, as long as in forewings, ending on posterior wing margin 5.8 mm from its base; area between CuAa and MP with one-two rows of cells, 0.9 mm wide; distal of end of CuAa, area between MP and posterior wing margin very long and broad; MP nearly straight, reaching posterior margin well distal of nodus level; MAa parallel with MP, nearly straight in its basal part, postdiscoidal area mm wide, narrower distally; 14 cross-veins in area between RA and RP, between arculus and nodus; base of RP3/4 2.7 mm distal of arculus, closer to arculus than to nodus; base of IR2 close to that of RP3/4, 4.9 mm distally; no antefurcal cross-vein in space between RP and MA basal of midfork (base of RP3/4); nodus oblique with subnodus aligned; more than seven postnodal cross-veins between C and RA and postsubnodal cross-veins between RA and RP1 not aligned with postnodals; pterostigmal brace probably not preserved; at least three cells below pterostigma; pterostigma sclerotized, long, 3.9 mm long, 0.8 mm wide; RP2 aligned with subnodus; oblique vein ‘O’ 4.3 mm and five cells distal of base of RP2; IR2 and RP2 nearly straight; area between MA and RP3/4 wider distally; area between RP3/4 and IR2 broadening distally; area between IR2 and RP2 with one row of cells and wider distally; several long intercalary veins between IR1 and RP1, and between IR1 and RP2 (but IR1 not clearly discernible), as well as between RP3/4 and IR2, and between IR2 and RP2 (these intercalaries are visible along posterior wing margin and parallel to the main longitudinal veins and have no apparent origin on them, but originate in the cross-venation).

##### Etymology.

Named after Daohugou Village, from where the specimen was collected.

##### Type locality and horizon.

Jiulongshan Formation, Middle Jurassic (Bathonian-Callovian boundary interval, ca 164–165 Ma); near Daohugou Village, Wuhua Township, Ningcheng County, Inner Mongolia, China.

## Discussion

The hindwing subdiscoidal cell enlarged and with a bulged posterior margin, correlated with a unique course of the anal vein AA ("pseudo-anal-loop"), which is strongly bent towards the posterior wing margin at the CuP-crossing, is the main apomorphy of the Isophlebioptera Bechly, 1996. The RP2 aligned with subnodus excludes affinities with the Parazygoptera Bechly, 1997 (= Sphenophlebiidae Bechly, 1997 + Euparazygoptera Bechly, 1997), even if the absence of antefurcal crossvein in the space between RP and MA basal of midfork would be an apomorphy with the Euparazygoptera (= Asiopteridae Pritykina, 1968 + Triassolestoidea Tillyard, 1918). Within this last clade, affinities with the Asiopteridae are excluded because IR2 is not zigzagged. The Triassolestoidea (Cyclothemistidae Bechly, 1996 and Triassolestidae Tillyard, 1918) are also excluded because the subnodus is well aligned with nodus. Affinities with the Selenothemistidae Handlirsch, 1939 are excluded because the hindwing distal side (MAb) of the discoidal cell is shorter than twice as long as the basal side; the postdiscoidal space is very narrow; and the hindwing subdiscoidal cell has a different shape, not posteriorly rounded (Nel et al. 1993). Affinities with the Architemistidae Tillyard, 1917 are excluded because the crossveins in the hindwing postdiscoidal space are not very long and oblique, some antenodal crossveins are present between anterior wing margin and ScP, and the shape of the hindwing subdiscoidal cell is different.

The gaff is not as long as in the Isophlebioidea Handlirsch, 1906, except some species with reduced venation (*Zygokaratawia reni* Nel et al., 2008) (Nel et al. 2008). Furthermore *Sinoeuthemis daohugouensis* shares with the Campterophlebiidae Handlirsch, 1920 the forewing discoidal cell basally opened but not the presence of antenodal crossveins between anterior wing margin and ScP (Li et al. 2012a, b), while the comparison with Isophlebiidae Handlirsch, 1906 is just opposing, i.e. *Sinoeuthemis* contradicts to Isophlebiidae in the basally closed forewing discoidal cell, but they are sharing the character (except in *Walleria*) presence of antenodal crossveins between anterior wing margin and ScP(Nel et al. 2009).

The only remaining isophlebiopteran family is the Euthemistidae Pritykina, 1968 (*Euthemis* Pritykina, 1968, forewing characters only known). *Sinoeuthemis* shares with this group the following potential synapomorphies: ‘several long intercalary veins between IR1 and RP1, and between IR1 and RP2, as well as between RP3/4 and IR2, and between IR2 and RP2 (these intercalaries are parallel to the main longitudinal veins and have no apparent origin on them, but originate in the cross-venation)’. Such intercalaries are also present in the parazygopteran family Sphenophlebiidae Bechly, 1997 (*Sphenophlebia interrupta* Bode, 1953, *Mesoepiophlebia veronicae* Nel & Henrotay in Nel et al. 1993 and *Ensphingophlebia undulata* Bode, 1953, plus maybe *Proeuthemis pritykinae* Nel & Jarzembowski, 1996). Nevertheless the Sphenophlebiidae have their RP2 not aligned with subnodus, as already indicated above. Also the better known representatives of the Sphenophlebiidae (*Mesoepiophlebia* and *Proeuthemis*) have no secondary antenodal crossveins between anterior wing margin and ScP, unlike *Sinoeuthemis*, while this character remains uncertain in *Ensphingophlebia* and *Sphenophlebia* because the original descriptions of Bode (1953) remain doubtful. On the contrary, *Sinoeuthemis* and *Euthemis* share the presence of these antenodal veins and a non-petiolated forewing. A further apomorphy of *Euthemis*, an ‘extremely narrow postdiscoidal area’, is more uncertain in *Sinoeuthemis*, even if the preserved part of forewing postdiscoidal area suggests that it should be present in our fossil.

The structure of the hindwing subdiscoidal cell and vein AA in *Sinoeuthemis* is quite similar to that of *Mesoepiophlebia*, except that vein CuAb makes a strong angle with AA in the former but these veins are nearly aligned in the later genus. These structures are unknown in *Euthemis* as there is no hindwing reported. The greatest difference between *Sinoeuthemis* and *Euthemis* is the short and simple CuAa in the former while this vein is quite long with numerous posterior branches in the later. *Proeuthemis* has also a shortened CuAa but RP2 not aligned with subnodus and wings with petiole.

So it remains that *Sinoeuthemis* should be placed close to the Euthemistidae rather than to the Sphenophlebiidae, even if it shows a mixture of characters alternatively present in different genera in these two families.

## Supplementary Material

XML Treatment for Euthemistidae

XML Treatment for Sinoeuthemis

XML Treatment for Sinoeuthemis daohugouensis
